# A case of demand ischemia from phendimetrazine

**DOI:** 10.1186/1757-1626-1-105

**Published:** 2008-08-18

**Authors:** Danny Landau, Judith Jackson, Gisselle Gonzalez

**Affiliations:** 1 Doctors Walk in Clinic, Department of Medicine, 500 North Westshore Blvd Suite 900 Tampa, Florida, 33609, USA; 2 Virus Hunter Inc., 3425 Collins Ave, Miami Beach Florida, 33140, USA

## Abstract

**Introduction:**

Phendimetrazine is a medication currently being used to help patients with weight loss. It shares a chemical structure with amphetamines. As such, it shares some of the same toxicities, which can include cardiac toxicity. This case highlights this principle.

**Case presentation:**

a 54 year old Caucasian female presented to our urgent care facility with complaints of chest pains and other symptoms suggestive of acute coronary syndrome. Ultimately, she was transferred to the emergency room. After evaluation there, it appeared she was having demand ischemia from prescription diet pills

**Conclusion:**

This case report demonstrates the potential dangers of amphetamine based diet pills. There have been other cases of cardiomyopathies related to phendimetrazine, but it is something that is rarely recognized in an outpatient setting. A case such as this demonstrates the importance of obtaining a careful medication history in all patients and in recognizing diet pills with an amphetamine base can cause cardiac toxicity.

## Case presentation

**Figure 1:**
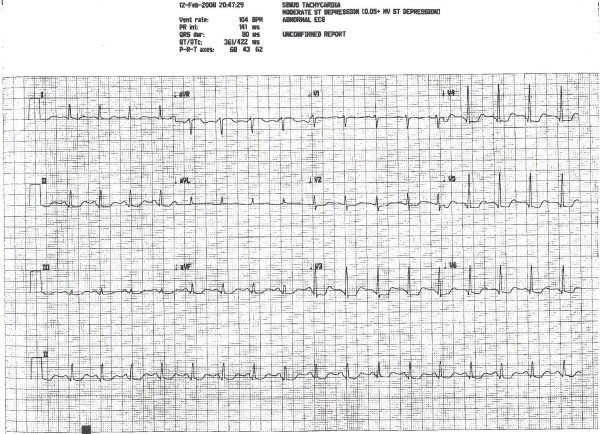
**An EKG taken from the patient while they were having chest pain.** It demonstrates T wave depression in lateral leads.

A 54 year-old Caucasian female presented to our urgent care facility complaining of nausea and vomiting, sense of impending doom and vague chest pain radiating toward her left side for about five hours. She never had similar symptoms in the past. She also denied anything that could have precipitated these symptoms. Her only past medical history was significant for spina bifida. Her medications included occasional Fiorinal (unknown dose), Xanax 0.5 mg as needed, and Phendimetrazine (unclear dose). Her social history was significant for smoking 1/2 pack per day cigarette use. She denied alcohol use. Family history was non contributory. She worked from home. Her physical exam showed a tachycardia of around 100 beats per minute, respiratory rate of 16, temperature of 98.1, and O2 saturation of 100% on room air. She was approximately 5'7" and 145 pounds. In general, she was an anxious appearing, diaphoretic woman in moderate distress, she had no elevated JVD at 30 degrees, her heart was tachycardic, but otherwise without murmur, gallops, or rubs, her lungs were clear, abdomen soft, and she had no peripheral edema. An EKG was checked which appears below (figure 1). After examination, there was concern for acute coronary syndrome (ACS). She was given nitroglycerin with relief of her chest discomfort. She was also given aspirin to chew. EMS was called and she was transferred to a local emergency room. She was hospitalized there for three days and after her discharge, we got permission from her to request records. While hospitalized, she was ruled out for ACS with negative troponins. She was also given beta blockade which resolved her tachycardia and her T wave changes on EKG. The next morning, she had an adenosine stress test which revealed normal uptake with no areas of ischemia and an ejection fraction of 55%. She was monitored for one more day and then discharged with instructions to discontinue her diet pills.

## Discussion

Phendimetrazine is a medication currently being used for weight loss, with potential for illicit use. It has a similar chemical composition of amphetamines, which is thought to account for its clinical actions [1]. Amphetamines are well recognized as an etiology of cardiac ischemia, however phendimetrazine is more rarely described in the literature as causing cardiac events. [2,3]. Acute effects include hyperpyrexia, mydriasis, chest pain, arrhytmias, delirium, and, rhabdomylosis, among others [2]. Long term use has been associated with dilated cardiomyopathies, some of which have resolved with discontinuation of the medication [3]. In this particular case, it appears she may have developed a demand ischemia from the medication. It is not known how much of the drug she was taking. Initially, she was resistant to accepting that phendimetrazine could induce side effects, and there was suspicion that she could have been taking more of the drug that recommended. In addition, she was not prescribed the medication and would not admit to where she obtained it. As the public seems to have more focus on using medications to induce weight loss, this may be a more recognized complication and heart conditions should likely be monitored prior to starting amphetamine based weight loss pills.

## Conclusion

Due to potentially detrimental effects of this medication, phendimetrazine should be used cautiously in many situations. As it shares its chemical structure with amphetamines, it also shares many of the side effects and the potential for abuse/addiction. There have been other reports in literature describing adverse outcomes from phendimetrazine as well as other weight loss medications. Therefore, cautious use is warranted.

## Abbreviations

ACS: Acute Coronary Syndrome.

## Competing interests

The authors declare that they have no competing interests.

## Authors' contributions

DL, JJ, GG have all been involved in and approve of the writing of this case presentation.

## Consent

Written informed consent was obtained from the patient for publication purposes. A copy can be obtained if requested by the Editor in Chief of this journal.
